# Performance of ChatGPT-4 as an Auxiliary Tool: Evaluation of Accuracy and Repeatability on Orthodontic Radiology Questions

**DOI:** 10.3390/bioengineering12101031

**Published:** 2025-09-26

**Authors:** Mercedes Morales Morillo, Nerea Iturralde Fernández, Luis Daniel Pellicer Castillo, Ana Suarez, Yolanda Freire, Victor Diaz-Flores García

**Affiliations:** 1 School for Doctoral Studies and Research, Universidad Europea de Madrid, 28670 Villaviciosa de Odón, Spain; mercedes.morales@universidadeuropea.es; 2 Department of Preclinical Dentistry II, Faculty of Biomedical and Health Sciences, Universidad Europea de Madrid, 28670 Villaviciosa de Odón, Spain; nerea.iturralde@universidadeuropea.es (N.I.F.); ana.suarez@universidadeuropea.es (A.S.); 3 Department of Health Sciences, Miguel de Cervantes European University of Valladolid, 47012 Valladolid, Spain; ldpellicer@uemc.es; 4 Department of Preclinical Dentistry I, Faculty of Biomedical and Health Sciences, Universidad Europea de Madrid, 28670 Villaviciosa de Odón, Spain; victor.diaz-flores@universidadeuropea.es

**Keywords:** artificial intelligence, orthodontics, ChatGPT, diagnostic accuracy, multimodal large language models (LLMs)

## Abstract

**Background:** Large language models (LLMs) are increasingly considered in dentistry, yet their accuracy in orthodontic radiology remains uncertain. This study evaluated the performance of ChatGPT-4 on questions aligned with current radiology guidelines. **Methods:** Fifty short, guideline-anchored questions were authored; thirty were pre-selected a priori for their diagnostic relevance. Using the ChatGPT-4 web interface in March 2025, we obtained 30 answers per item (900 in total) across two user accounts and three times of day, each in a new chat with a standardised prompt. Two blinded experts graded all responses on a 3-point scale (0 = incorrect, 1 = partially correct, 2 = correct); disagreements were adjudicated. The primary outcome was strict accuracy (proportion of answers graded 2). Secondary outcomes were partial-credit performance (mean 0–2 score) and inter-rater agreement using multiple coefficients. **Results:** Strict accuracy was 34.1% (95% CI 31.0–37.2), with wide item-level variability (0–100%). The mean partial-credit score was 1.09/2.00 (median 1.02; IQR 0.53–1.83). Inter-rater agreement was high (percent agreement: 0.938, with coefficients indicating substantial to almost-perfect reliability). **Conclusions:** In the conditions of this study, ChatGPT-4 demonstrated limited strict accuracy yet substantial reliability in expert grading when applied to orthodontic radiology questions. These findings underline its potential as a complementary educational and decision-support resource while also highlight its present limitations. Its role should remain supportive and informative, never replacing the critical appraisal and professional judgement of the clinician.

## 1. Introduction

Artificial intelligence (AI) is increasingly embedded in dentistry and, specifically, orthodontics, supporting diagnosis, planning, and patient communication [1]. This field benefits from advanced systems that replicate human processes such as learning, reasoning, and decision-making [2,3]. Large language models (LLMs) are AI systems trained on extensive datasets collected from diverse online sources, enabling them to generate texts, images, and other types of content with realistic and human-like characteristics [4].

Machine learning (ML) is a fundamental branch of AI that enables systems to learn from data, identify patterns, and make predictions without explicit programming. Within ML, deep learning (DL) represents an approach based on deep neural networks, which are capable of autonomously extracting complex features from data [5]. In orthodontics, these technologies have significantly enhanced diagnostic accuracy, treatment planning, and patient management [6,7,8].

The recent incorporation of automated machine learning (AutoML) platforms allows orthodontists to create complex predictive models for decision-making, facilitating the clinical adoption of AI by automating complex steps with the potential to improve the quality and efficiency of orthodontic treatment [9]. One of the most significant advances is the use of automated tools to analyze cephalometric radiographs. Through neural networks, the identification and annotation of cephalometric landmarks have become faster, more accurate, and more consistent–sometimes matching the quality of expert manual tracing. In addition, these tools can be used to predict bone growth, optimize the use of temporary anchorage devices (TADs), and assist in surgical planning for more complex orthodontic treatments [10]. This not only saves time on repetitive tasks but also reduces human error and enhances diagnostic standardization [11,12].

Such innovations are transforming clinical orthodontics by enabling more personalised patient care [13]. Remote monitoring powered by AI is reshaping teleorthodontics, allowing for more continuous and detailed follow-up without requiring the patient’s physical presence in the clinic [14].

In healthcare, models like ChatGPT have also proven useful in medical education and patient communication [15]. These tools are capable of generating evidence-based responses to complex questions in a clear and comprehensive manner [16].

However, the implementation of AI in orthodontics also presents important challenges and limitations. Concerns have been raised regarding its practical applicability in clinical contexts, as well as the ethical implications of deploying LLMs in healthcare settings [15]. Among these ethical concerns is the potential for bias, particularly when training datasets are disproportionately composed of specific demographic groups [17]. These issues highlight the need for further research and the development of ethical frameworks to guide the clinical integration of such technologies [15,18,19].

LLMs such as ChatGPT have shown potential in generating real-time responses, supporting clinical decision-making and patient education [20,21,22]. Prior studies have evaluated ChatGPT’s performance in areas like prosthodontics and oral surgery, identifying both its strengths in clinical explanation and its limitations in accuracy and consistency [20,23]. Nevertheless, the reliability and precision of these responses in the context of orthodontic radiodiagnosis have not yet been extensively investigated.

The aim of this study is to evaluate the diagnostic accuracy and reproducibility of ChatGPT’s responses to open-ended questions concerning orthodontic radiological diagnosis, by comparing them to established clinical standards.

## 2. Materials and Methods

According to the terms of the Declaration of Helsinki, this research did not require ethical approval as human participants were not involved.

### 2.1. Study Design

To assess the accuracy and repeatability of the answers provided by ChatGPT-4 to questions regarding orthodontic radiodiagnosis, 50 open-ended short questions were developed by two experienced authors (A.S., Y.F.) with reference to guidelines published by the British Orthodontic Society, the American Academy of Oral and Maxillofacial Radiology, the European academy of Paediatric Dentistry and The European Commission [24,25,26,27]. Two experienced orthodontists (M.M.M. and N.I.F.) independently assessed each question based on relevance for routine diagnosis/planning and coverage of common decision points (e.g., panoramic vs. CBCT indications, skeletally related imaging). The relevance of each question was assessed using a 3-point Likert scale (0 = disagree; 1 = neutral; 2 = agree). Any discrepancies were reviewed by a third expert independently (L.D.P.C.). As a result of this process, 30 questions were selected (Table 1).

**Figure 1:**
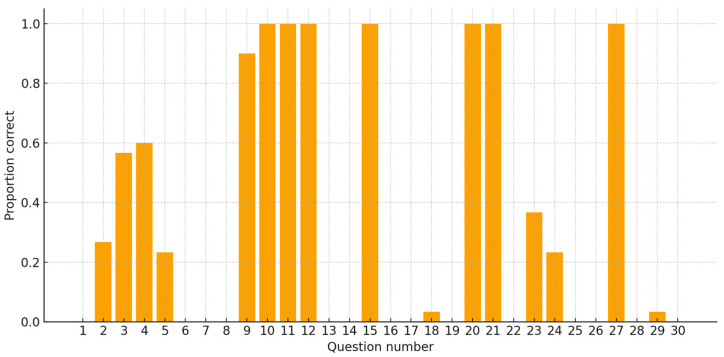
Proportion of correct (grade = 2) answers for each of the 30 orthodontic radiology questions (n = 30 repetitions per item; total 900 answers).

Responses were generated in March 2025 using the ChatGPT web application with the “GPT-4” model label (OpenAI) using two accounts (M.M.M.; V.D.G.). To enter the questions, a specific prompt was designed. This specific prompt was designed with two main objectives: first, to simulate a realistic clinical interaction between an orthodontist and a general dentist; and second, to elicit concise and specific responses, thereby enabling a more objective and standardized evaluation by the expert panel. Therefore, the prompt used was as follows: “Imagine you are an orthodontist and I am a general dentist. Please answer the following question: (QUESTION) (only short answer).” (Figure 1) The rationale was to simulate a brief clinician-to-clinician exchange and elicit concise, scorable statements over discursive essays. In order to reduce any possible memory bias, each answer was collected in a new chat and sampled three times of day (morning/afternoon/evening). The responses were catalogued in an Excel© spreadsheet (Microsoft, Redmond, Washington, DC, USA). The answer generation process took place in March 2025.

### 2.2. Evaluation Criteria

The 900 answers generated were evaluated independently by two authors (N.I, LD.P.C) using a 3-point Likert scale (0 = incorrect; 1 = partially correct or incomplete; 2 = correct) (Table 2) and with access to the different guidelines used. Graders were provided with a written reference key consolidating guideline-anchored expectations per item. Any discrepancies between the two evaluations were resolved by an independent assessment from a third senior experienced author (V.D.G). For each of the 30 questions, the relative frequency (n) and absolute percentage (%) of answers graded as 0 (incorrect), 1 (incomplete or partially correct), and 2 (correct) were described.

### 2.3. Statistical Analysis

To assess the performance of ChatGPT, the accuracy and repeatability values were calculated. In order to analyze the accuracy of the answers generated by ChatGPT, the proportion of questions to which an answer of grade 2 (correct) was given was calculated for the total answers in the question set and for each individual question. This calculation was based on the Wald binomial method and took into account the 95% confidence interval.

The assessment of repeatability was conducted through concordance analyzes weighted for ordinal categories and multiple repetitions of the gradings given by the experts (including percentage agreement, Brennan and Prediger coefficient, Conger generalized Cohen kappa, Fleiss kappa, Gwet AC, and Krippendorff alpha) along with their corresponding 95% confidence intervals. The estimated coefficients were classified according to the benchmark scale proposed by Gwet in 2014 [28]. All of the statistical analyzes were carried out using a statistical software program (STATA version BE 14; StataCorp, College Station, TX, USA).

## 3. Results

A total of 900 answers were generated by ChatGPT, providing 30 responses for each of 30 questions. Table 3 shows the absolute and relative frequencies of the expert’s grading for each answer. To illustrate this heterogeneity more clearly, the proportion of correct (grade = 2) answers per question is displayed in Figure 1.

The range of percentage of correct repetitions was from 0% to 100%, depending on the specific question. The overall accuracy of the answers generated by ChatGPT was 34.1%, with a 95% confidence Interval ranging from 31.0% to 37.2%.

Among the questions asked, fifteen exhibited 100% repeatability, yielding the same score across all 30 answers of each of these questions. Four of the questions (7, 8, 13 and 30) indicated a 100% inaccuracy rate, as all answers generated were graded as incorrect. In contrast, questions 10, 11, 12, 15, 20, 21 and 27 exhibited a 100% rate of repeatability and accuracy with all responses correct. A total of 15 questions showed variability in terms of their degree of repeatability.

**Figure 2:**
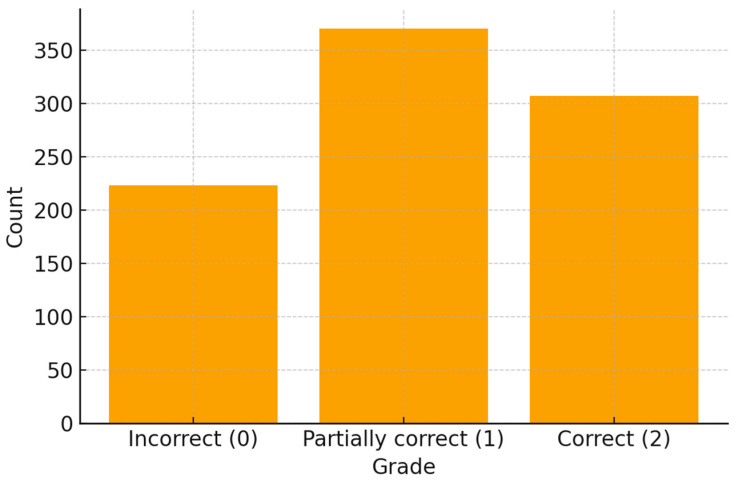
Overall distribution of expert grades across 900 ChatGPT-4 answers (0 = incorrect; 1 = partially correct; 2 = correct).

The results showed a substantial repeatability according to the benchmark scale used: <0.0 Poor, 0.0–0.2 Slight, 0.2–0.4 Fair, 0.4–0.6 Moderate, 0.6–0.8 Substantial, 0.8–1.0 Almost Perfect (Table 4). This overall pattern of performance is summarised in Figure 2.

## 4. Discussion

According to the results obtained, ChatGPT-4 achieved limited strict accuracy (34.1%) on text-only orthodontic radiology questions, with a significant degree of variability among questions and an assessment reliability rated as substantial to almost perfect. These findings suggest that, although ChatGPT may produce correct responses in certain contexts, question formulation and content domain strongly influence performance.

A pronounced heterogeneity was observed in the percentage of correct answers depending on the specific question. Among the 30 questions, 14 (46.7%) yielded no correct answers, while 10 (33.3%) surpassed a 50% correctness rate. The remaining 6 questions (20.0%) fell within an intermediate accuracy range, with correct responses between 1% and 50%. These results indicate that the model’s performance varied substantially based on the specific content evaluated.

The variability in results across different dental specialties is noteworthy. In this study, ChatGPT achieved an overall accuracy of 34.1% in the field of orthodontic radiodiagnosis. In contrast, in oral surgery, the tool achieved an accuracy of 71.7%, positioning it as a system with remarkable capabilities for information processing and understanding, and with consistency levels ranging from moderate to almost perfect [20]. Contrary to the findings of the present study, ChatGPT’s performance in endodontics has higher accuracy, with a rate of 57.3% and a consistency rate of 85.4% when answering binary questions [29]. In prosthodontics, despite using the same sample size, the model achieved only 25.6% accuracy, suggesting that the accuracy and repeatability of ChatGPT’s responses are influenced by the question format and the technical complexity [23]. While in paediatric dentistry [30], ChatGPT-4 achieved the highest average score (8.08 out of 10), highlighting a stark contrast with our findings. Therefore, it can be observed that different studies have reported varying accuracies depending on the specialty.

Another possible explanation for the discrepancy among studies may lie in the study design, as some studies evaluated only one answer per question. This methodology may constrain the interpretation of the findings, as relying on a single answer increases the risk of bias, particularly relevant given the phenomenon whereby large language models generate outputs that appear coherent and reliable but ultimately lack factual accuracy or clinical relevance [31]. In addition, the literature shows that specific and well-defined questions tend to yield better answers, while more open-ended or clinically demanding questions decrease the quality, a trend also observed in the results of the present study. 

Regarding the model assessed, Makrygiannakis et al. [16] found that Microsoft Bing Chat outperformed ChatGPT-4 in orthodontics (7.1 vs. 4.7), followed by Google Bard (4.8) and ChatGPT 3.5 (3.8). These inter-specialty differences may be attributable to factors such as training dataset composition and the varying complexity of paediatric dentistry versus orthodontics.

A relevant point of comparison is the study by Tanaka et al. [10], which specifically assessed ChatGPT-4 in orthodontics, focusing on clear aligners, temporary anchorage devices (TADs), and digital imaging. That study reported that 71.6% of the responses were rated as “very good” and 15.1% as “good,” with median scores of 5.0 across all domains. However, the inter-rater agreement in that study was low (Fleiss’s Kappa = 0.004), suggesting considerable subjectivity. In contrast, our study demonstrated high inter-rater reliability, with 95.6% agreement between the two main reviewers (860 out of 900 responses), requiring a third grader in only 4.4% of cases. Weighted concordance analyses confirmed this consistency: agreement rate 0.938 (95% CI: 0.911–0.965), classified as “almost perfect”; Brennan & Prediger kappa index 0.833 (95% CI: 0.759–0.907); and Cohen/Conger kappa 0.813 (95% CI: 0.713–0.913), both interpreted as “substantial.” These metrics support the confidence in the accuracy of the evaluations and reinforce the reliability of our findings.

When comparing our results to those of Naureen and Kiani [32], who reported 92.6% average accuracy for ChatGPT-4 in orthodontic diagnosis and treatment, our findings reveal substantially lower performance (34.1%). However, their methodology relied on a single response per item evaluated with a five-point qualitative scale, whereas our design incorporated 30 repetitions per question, allowing us to assess not only accuracy but also response variability and model reliability. Cross-study comparisons remain difficult due to methodological differences and the heterogeneity of specialties assessed.

Recent research has begun comparing ChatGPT versions, particularly ChatGPT-4 versus ChatGPT-4o, with the latter showing improved performance in clinical contexts and lower rates of hallucinated responses [33,34]. However, applied to frequently asked orthodontic questions, the responses still fall short of expert-level knowledge [35]. Thus, continued refinement is necessary, particularly through strategies that promote clearer, more contextually grounded answers.

A potential limitation of this study is the use of a directed prompt. It has been observed that the prompt employed can influence the responses generated. Therefore, the use of a prompt oriented toward a specific answer could have conditioned the results obtained. For this reason, it is essential to conduct further studies analyzing the performance of ChatGPT in different contexts and with diverse prompts, which would allow for a broader exploration of the model’s capabilities and limitations [36].

It is also important to recognise the limitations identified in this study. In addition to the influence of question complexity on performance and reliability, the restricted number of items assessed may not capture the full range of orthodontic scenarios, and the simulated setting with standardised prompts may differ from real clinical interactions. Although inter-rater agreement was high, some degree of subjectivity in expert grading cannot be entirely excluded, and the model’s outputs remain sensitive to prompt formulation. Finally, as ChatGPT-4 itself is not specifically trained on orthodontic datasets, its accuracy and applicability to current clinical practice may be limited.

Considering the rapid evolution of these tools and their considerable potential, it is crucial to enhance their alignment with clinical reasoning and specialty-specific expertise. Current evidence does not support using these tools as a substitute for professional judgement. Furthermore, future studies should investigate the potential of ChatGPT as an auxiliary diagnostic tool in orthodontic radiology, employing robust and clinically realistic protocols. It is also important to recognise the limitations identified in this study, particularly the impact of question complexity on the model’s performance and reliability.

## 5. Conclusions

In the conditions of this study, ChatGPT-4 demonstrated limited strict accuracy yet substantial reliability in expert grading when applied to orthodontic radiology questions. These findings underline the model’s potential as a complementary educational and decision-support resource, while highlighting its present limitations. At this stage, its role should be regarded as supportive and informative, offering auxiliary guidance that may stimulate reflection or learning but never replacing the critical appraisal and professional judgement of the clinician. 

## Figures and Tables

**Table 1:** Questions selected for answer generation by ChatGPT-4.

Question Number	Question Description
1	What is the function of panoramic radiography in orthodontics?
2	Do we need to take an orthopantomography before the clinical examination for a correct diagnosis in orthodontics?
3	Do we need for a correct diagnosis in orthodontics to take a CBCT before the clinical examination?
4	In what type of radiographs can we be able to assess the state of skeletal maturation of the patient?
5	What radiological tests would help in the diagnosis and treatment of impacted teeth?
6	In patients with mixed dentition, is it indicated to routinely take a panoramic radiograph to assess tooth replacement?
7	What radiographs should be taken at the end of an orthodontic treatment?
8	Is it indicated to take post-treatment x-rays at the end of orthodontic cases?
9	If third molars need to be extracted for orthodontic treatment, which radiodiagnostic test is the most indicated prior to extraction?
10	Is pre-treatment lateral skull teleradiography indicated in patients under 10 years of age with class III?
11	Is pre-treatment lateral skull teleradiography indicated in patients under 10 years of age with Class II?
12	Is it appropriate to perform lateral skull radiography in patients between 10 and 18 years of age who are about to start treatment with functional appliances?
13	Is it indicated to take radiographic records in a patient older than 10 years if the canines have not erupted, but by palpation we can intuit that they are in a favourable position?
14	Is it considered necessary to take an X-ray in a patient over 14 years of age who has not erupted the second permanent molars?
15	Is a lateral skull X-ray indicated before starting treatment in patients over 18 years of age with biprotrusion?
16	How should we position the patient to take a correct lateral skull teleradiography?
17	How should we prepare the patient to take a correct panoramic radiograph?
18	How should we instruct the patient to bite in order to take a panoramic X-ray correctly?
19	In a patient with symptoms associated with temporomandibular dysfunction, is it necessary to take an orthopantomography for a correct diagnosis?
20	Is it correct to perform a CBCT in patients with temporomandibular dysfunction if we want to know the position of the articular disc?
21	What test should we perform to assess the condition of the articular disc of the temporomandibular joint?
22	What should the decision to perform a CBCT on an orthodontic patient be based on?
23	What are the indications for CBCT in orthodontics?
24	In which cases is it justified to perform a CBCT on an orthodontic patient?
25	Can a cephalometric study be performed using the image obtained from a CBCT?
26	What is the recommended field of view (FOV) if it is necessary to perform a CBCT on an orthodontic patient?
27	What radiological test could you perform to measure bone volume when placing a mini-screw (TAD)?
28	Is the use of CBCT routinely indicated in case planning where TADS are necessary?
29	When would a CBCT be justified when extracting a supernumerary tooth?
30	In which situation would a CBCT be indicated and justified for the evaluation of an impacted tooth?

**Table 2:** Grading System used for ChatGPT Answers.

Grading	Grading Description
Incorrect (0)	The answer provided is completely incorrect or unrelated to the question. It does not demonstrate an adequate understanding or knowledge of the topic.
Partially correct or incomplete (1)	The answer shows some understanding or knowledge of the topic, but there are significant errors or missing elements. Although not completely incorrect, the answer is not sufficiently correct or complete to be considered certain or adequate.
Correct (2)	The answer is completely accurate and shows a solid and precise understanding of the subject. All major components are addressed in a thorough and accurate manner.

**Table 3:** Distribution of experts grading for ChatGPT answers.

	Incorrect		Partially Correct or Incomplete		Correct	
Question	n	Percentage (%)	n	Percentage (%)	n	Percentage (%)
1	17	56.67	13	43.33	0	0.00
2	0	0.00	22	73.33	8	26.67
3	2	6.67	11	36.67	17	56.67
4	0	0.00	12	40.00	18	60.005
5	0	0.00	23	76.67	7	23.33
6	20	66.67	10	33.33	0	0.00
7	30	100.00	0	0.00	0	0.00
8	30	100.00	0	0.00	0	0.00
9	0	0.00	3	10.00	27	90.00
10	0	0.00	0	0.00	30	100.00
11	0	0.00	0	0.00	30	100.00
12	0	0.00	0	0.00	30	100.00
13	30	100.00	0	0.00	0	0.00
14	0	0.00	30	100.00	0	0,00
15	0	0.00	0	0.00	30	100.00
16	5	16.67	25	83.33	0	0.00
17	0	0.00	30	100.00	0	0.00
18	29	96.67	0	0.00	1	3.33
19	4	13.33	26	86.67	0	0.00
20	0	0.00	0	0.00	30	100.00
21	0	0.00	0	0.00	30	100.00
22	6	20.00	24	80.00	0	0.00
23	0	0.00	19	63.33	11	36.67
24	0	0.00	23	76.67	7	23.33
25	0	0.00	30	100.00	0	0.00
26	20	66.67	10	33.33	0	0.00
27	0	0.00	0	0.00	30	100.00
28	0	0.00	30	100.00	0	0.00
29	0	0.00	29	96.67	1	3.33
30	30	100.00	0	0.00	0	0.00

**Table 4:** Repeatability assessment for 30 repetitions of 30 questions generated by ChatGPT, based on expert grading.

Methods	Coeficient	95% Confidence Interval	Benchmark Scale
Percent Agreement	0.938	0.911–0.965	Almost perfect
Brennan and Prediger	0.833	0.759–0.907	Substantial
Cohen/Conger’s Kappa	0.813	0.713–0.913	Substantial
Scott/Fleiss’ Kappa	0.813	0.768–0.909	Substantial
Gwet’s AC	0.838	0.713–0.913	Substantial
Krippendorff’s Alpha	0.813	0.911–0.965	Substantial

Benchmark scale used: <0.0 Poor, 0.0–0.2 Slight, 0.2–0.4 Fair, 0.4–0.6 Moderate, 0.6–0.8 Substantial, 0.8–1.0 Almost Perfect.

## Data Availability

The relevant information for this article is available on the Open Science Framework repository (https://osf.io/k5tcp/?view_only=7fb04cfd0ca84b718cfadb2fb9c5cbcf, accessed on 20 September 2025).

## Abbreviations

The following abbreviations are used in this manuscript:

AI	Artificial Intelligence
LLMs	Large language models
ML	Machine Learning
DL	Deep learning
AutoML	Automated Machine Learning
TADs	Temporary Anchorage Devices
